# Editorial Extracts and Gleanings

**Published:** 1874-01

**Authors:** Swan M. Burnett

**Affiliations:** Tennessee


BY SWAN M. BURNETT, M.D., OF TENNESSEE.

*Influence of School Life on Vision.*—Dr. Agnew, of New York, (Medical and Surgical Reporter) says in reference to this matter: "Every practitioner who is called upon in our city to engage in ophthalmic practice, will testify that those forms of eye disease which are directly traceable to over or faulty use of the organ, are greatly on the increase. Of these maladies, near-sightedness may be taken as the type and example. Near-sightedness is increasing, and can be traced without the slightest difficulty to the influence of public and private schools. Such a statement, capable of easy proof, does not produce the impression upon the public mind that it should, because there is profound ignorance among the public as to the nature of near-sightedness. Most persons think a near-sighted eye a good one to have, that it is a 'strong eye'—one in which there is an economy of visual force not enjoyed by eyes that see well at long range as well as near at hand; that a near-sighted eye may cause some inconvenience to its possessor during his early life, to be compensated for, however, by his being able to dispense with convex glasses for vision after forty years of age."

A greater error, and one more pernicious to good vision, could hardly be entertained by the popular mind. In some exceptional cases, where the myopia is of low degree, there is a compensation for the loss of Accommodation in Presbyopia, by the slight increase of the antero-posterior axis of the eye commonly found in near-sightedness. These cases are rare, while, according to Denders, the law of Myopia is to a steady increase. Two-thirds or more of the myopics you meet tell you that their near-signtedness is worse than it was several years previous.

It should be borne in mind that the myopic eye is not a better one than a normal eye, but that it is not even as good, but is emphatically a diseased one, and should be cared for and treated as such. This subject has been noticed more at length in the Georgia Medical Companion for October, 1872.

The influence of school life in the production of this, together with other troubles of the organ of vision, has been treated of extensively by Dr. Leibreich, now of St. Thomas' Hospital, London. These diseases of the eye, either produced or aggravated during school life, and directly traceable to the imperfect arrangements of the school-rooms, are: 1. Decrease of the range of vision (Myopia.) 2. Decrease of acuteness of vision (Amblyopia.) 3. Decrease of endurance of vision (Asthenopia.)

The rules he lays down for obviating these troubles are these:

"The light must be sufficiently strong, and fall on the table from the left hand side, as far as possible from above. The children ought to sit straight, and not have the book nearer to the eye than ten inches at the least. Besides this, the book ought to be raised 20° for writing, and about 40° for reading." If these rules were strictly adhered to, we should fear much less complaint from our school children about their eyes, and see fewer glasses on the noses of our literary friends on the sunny side of forty-five.

*New Incision for Linear and Traction of Cataract.*—In the Allgem. Wien, Med. Ztg., 1873—27, 28, 29, 30, 31—Jaeger gives a description of an improved instrument for making a more nearly linear incision for cataract extraction, and his manner of operating.

A perfectly linear incision, that is an incision in which all the points shall fall in the same ocular meridian, is most difficult of execution. Gräfe's so-called peripheric linear incision is not in reality linear, but on a flop with a small angle. Hence, even here the lips of the wound are liable to be forced apart by the intra-occular pressure and thus fail in union.

The instrument he now uses is a triangular knife, somewhat like the old Beers knife, but much narrower. It is, in the blade, 33 to 35 m m in length and 5½ to 6½ m m at the greatest breadth. The blade is blunt at the back, but thin in order to fit accurately in the angle of the wound.

The edge is curved. The surface of the blade is cylindrical, the concavity being in front with a radius of 6 or 7 m m, while the posterior surface is convex with a less radius of 5 m m. The operation is performed as follows: "The patient is placed either in the sitting or reclining position; the lids are kept apart by a simple holder which depresses the lower lid, or in very restless patients, the double speculum is used; the bulb is fixed by the operator who grasps a fold of the conjunctiva below the lower edge of the cornea with a blunt notched pincette. I place the knife with its concavity directed forwards, its edge upwards, parallel with the plane of the iris as to its length, and as to its surface, inclined to an angle of from 35° to 40° to the same, and pass it, turning it slightly forwards in a direction corresponding to the curve of its blade, or where possible, keeping it unaltered in a line to its back through the upper portion of the anterior chamber. Having made the puncture and counterpuncture, the bulb is no longer fixed by the pincette, but in proportion as the bulb tends to roll upwards, counter-pressure is made with the back of the knife."

According to Jaeger, the advantages of this method of operating lays—

1st. In the production of the most nearly linear wound possible, of a size sufficient for the largest cataract.

2d. In the possibility of fixing the bulb as desired during the whole operation.

3d. In the more or less total retention of the aqueous fluid until the completion of the incision.

4th. In the relatively slight increase of the intra-ocular pressure during the accomplishment of the incision.

5th. In relatively easy escape of the cataract.

6th. In subjecting the patient to an after treatment neither tedious nor exhausting.

In distinction from the other linear incisions he calls this one the "concave incision."—(Dr. Hohlsnitt.)

*Destruction of the Eye by Herpes Zoster Ophthalmicus.*—Dr. B. Joy Jeffries, one of the most active and energetic ophthalmologists of Boston, communicated to the last meeting of the American Ophthalmological Society, two interesting cases of the above affection, which resulted in loss of vision and destruction of the eye. He mentions three reported cases in which death has resulted from ophthalmic shingles. In one case a post-mortem was made and inflammation of the Gasserian ganglion was found, thus corroborating the assertion put forth in 1861 by Barensprung, "that zoster depends on a disease of the ganglionic system, and in special cases on irritation of some one of the special ganglia or Gasserian; yet the peripheric irritation of a nerve having ganglionic fibers in it may be followed by a limited eruption of zoster vesicles." The pathology of zoster then seems to be pretty well settled, and it must take its place by right among the neuroses.

The conclusions of Dr. A. Hybard, who recently published a monograph on the subject of ophthalmic zoster are given as follows: 1. Ophthalmic zona is an herpetic eruption developed over the territory of the first branch of the trigeminal. 2. Ocular alterations coëxist with the cutaneous eruption, the most important of which are keratitis and iritis. These may exist together or singly; keratitis more frequent than iritis. 3. Zona is the cutaneous expression of irritation or inflammation of distinct parts of the nervous system (mixed nervous trunk, sensitive trunk, spinal ganglion, posterior sensitive root, posterior columns of the cord.) 4. Ophthalmic zona is the expression of irritation or inflammation of the first trigeminal branch.

The process causing the eruption may be developed in the Gasserian ganglion, or in the track of the ophthalmic branch. 5. The lesions of the cornea and iris belong to the same class of phenomena as the cutaneous, "they are due to irritation, or inflammation of the ciliary twigs of the nasal branch of the ophthalmic division. Most often they correspond to the distribution of the eruption in the cutaneous territory of the nasal nerve. 6. The cutaneous eruptions and the ocular lesions are not dependent on the paralysis of the vaso-motors and consecutive neuro-paralytic hyperæmia. They must be referred to the direct influence of the nervous system on nutrition. Of all the theories suggested, the one seems most satisfactory and simple, which attributes these trophic troubles to irritation of the trophic nerves."

Dr. Jeffries also contributed two cases of intra-ocular tumor—sarcoma of the cheroid—in which enucleation was performed, and a case of "traumatic rupture of the cheroid, without dirrct injury to the eye."

*Examination of Nasal and Aural Cavities and Application to Them.*—We have received from Thomas F. Rumbold, M.D., of St. Louis, a small pamphlet describing "new instruments for examinations and applications to the cavities of the nose, throat and ear, and some remarks about the remedies that may be administered by means of them."

From his descriptions, most of the instruments are admirably adapted to the purposes for which he uses them. For instance, as a nasal speculum and dilator, he has a very simple but very efficacious contrivance. Instead of the bivalve aural speculum, such as is commonly used for the purpose of dilating the alæ nasi, he employs "two elastic hooks, one attached to each end of a piece of India rubber webbing, long enough to pass around the head. They are made out of India rubber hair-pins, bent into proper shape, after being softened by heat over a lamp. One is placed in each nostril, by compressing the extremities of the hooks. When this pressure is relaxed, the nasal openings are lengthened perpendicularly, and the traction of the rubber band draws the alæ wide apart." We have thus the nasal openings widely extended, while both hands of the operator are free to manipulate,

His "hinge pharyngeal mirror" is also a most convenient arrangement, allowing the position and angle of the mirror with the handle, to be changed without withdrawal of the instrument. The "uvula retractor" is also a convenient instrument, as is likewise the "spreading soft palate retractor." Both must be of very material assistance in making examinations of the posterior nares.

The "acou-otoscope" is but a modification of Siegle's apparatus, but at the same time made to serve the purpose of an auscultation tube. For making applications, the spray producers with modifications of the terminal ends of the glass tubes of such a character as to throw the spray in different directions, are used.

As remedies, he finds that, as cleansers of the cavities, nothing is so good as salt and water, ʒi. to the pint. In ozœna, to destroy offensive odor, he finds nothing so good as bromo-chloralum, in the strength of ℥ii. to the pint, or even stronger.

In acute and chronic inflammations, carbolic acid stands at the head of the list of remedies used. The following formula is a favorite one: "℞.—Acidi carbolici cryst., ℈i. to ℈ii.; glycerinæ, f℥i.; aquæ, f℥viii. M. Applied every day or every other day. Several other remedies are mentioned, as the geranium maculatum, muriate of ammonia, aconite and cupri sulphas, which latter he thinks superior to argt. nitratis in promoting healthy granulations in phagedenic ulceration.

*Episcloral Melanotic Sarcoma.*—Professor H. Knopp, of New York, presented to a late meeting of the New York Pathological Society a very unique and interesting pathological specimen of the above character. It was seated on the inner side of the sclerotic near the margin of the cornea, and presented the appearance of a small blackish elevation. It had made its appearance six years previously, and had been twice removed. The Professor enucleated the globe, and on microscopical examination found the histological characters to be small, spindle-shaped and roundish cells. In some places small, round, unpigmented cells were abundant. In six days after the enucleation, a tumor of similar appearance made its appearance on the inner side of the orbit, which was removed, and a microscopical examination showed the same characteristic cells.

This is the only instance of a malignant growth of this character to Doctor has ever seen in that locality. These tumors, whenever they make their appearance, should be removed thoroughly and completely, for their great tendency to spread makes it unsafe to leave any of the adjacent tissue, as this case shows.

*Night Sweats*, in connection with phthisis, empyema, etc., have been successfully treated in Bellevue Hospital with $\frac{1}{256}$-grain doses of sulphate of atropia at bed-time. It is usually in cinnamon water.—*New York Medical Record.*

